# Sex-Specific Differences in Neuromuscular Performance, Joint Mobility, and Postural Control in Elite Karate Athletes

**DOI:** 10.3390/s26103161

**Published:** 2026-05-16

**Authors:** Luca Molinaro, Massimo Montecchiani, Stefano Rossi, Juri Taborri

**Affiliations:** 1 Department of Theoretical and Applied Sciences, eCampus University, 22060 Novedrate, Italy; 2 FIJLKAM—Italian Federation of Judo, Wrestling, Karate and Martial Arts, 00100 Rome, Italy; m.montecchiani@gmail.com; 3 Department of Economics, Engineering, Society and Business Organization, University of Tuscia, 01100 Viterbo, Italy; stefano.rossi@unitus.it

**Keywords:** karate, sex differences, inertial sensors, monopodalic balance, joint mobility, jump

## Abstract

Elite karate performance depends on neuromuscular power, joint mobility, and postural stability, yet sex-specific biomechanical profiles in this population remain insufficiently characterized. This study examined differences in lower-limb explosive power, joint kinematics, and visual-dependent postural control in 28 international-level karatekas. Assessments included vertical jump tests, joint mobility tasks performed at preferred and maximum velocities, and stabilometric evaluations under eyes-open and eyes-closed conditions. Males demonstrated significantly longer flight and phase times across all jump tests (all *p* < 0.05), and higher maximum angular velocities during shoulder flexion and extension, while females exhibited greater hip abduction range of motion during the Wall Split test (all *p* < 0.05). In postural control, females showed larger Ellipse Area and Path Length under eyes-open conditions (*p* < 0.05), but these differences were eliminated when vision was removed. The Romberg Index indicated comparable reliance on visual input between sexes. These findings may support the development of more individualized, sex-specific conditioning strategies in elite karate.

## 1. Introduction

Karate is a widely practiced martial art characterized by high-intensity, intermittent actions requiring the integration of physical, physiological, and cognitive abilities [1]. At the elite level, athletes must execute rapid and explosive movements, including strikes, evasions, and multidirectional footwork, within very short time frames [2]. Consequently, performance primarily relies on three key functional components: lower-limb explosive power, joint mobility, and postural stability.

Lower-limb power is essential for rapid distance management and effective scoring techniques [3], while an adequate range of motion, particularly at the hip and shoulder joints, supports the execution of complex and high-amplitude movements [4]. Additionally, the dynamic and unpredictable nature of karate requires efficient postural control and visual–motor integration to maintain balance and quickly restore stability during both offensive and defensive actions [5].

Recent literature on karate biomechanics primarily focuses either on the kinematic analysis of specific gestures or on sports injury epidemiology. Regarding technical execution, studies on fundamental strikes (e.g., Gyaku-Tsuki and Mawashi-Geri) highlight that sequential joint activation, core stability, and lower-limb explosive power are crucial for maximizing impact force [6,7,8]. Parallel to performance optimization, research documents a high risk of injuries to the head, face, and lower extremities driven by the sport’s high-velocity, repetitive maneuvers [9,10]. To mitigate these sport-specific risks, targeted preventive methods emphasize improving neuromuscular control, joint mobility, and postural stability, demonstrating that biomechanical efficiency is equally vital for both athletic performance and injury prevention [11].

Beyond isolated gesture analysis, broader physical and physiological profiling of elite World Karate Federation (WKF) athletes has recently gained significant traction. As highlighted by a recent systematic review by Gaweł et al. [12], evaluating a combination of motor abilities including flexibility, agility, and lower-limb power, is essential to establish definitive performance benchmarks for elite kumite and kata athletes. In this context, standardized jump assessments, such as the Countermovement Jump (CMJ), Repeated Countermovement Jump (RCMJ) and Squat Jump (SJ), have been widely employed to evaluate neuromuscular readiness, stretch-shortening cycle (SSC) efficiency, and leg stiffness in martial artists [13,14,15].

Similarly, in the domain of postural control, posturographic analyses have consistently demonstrated that karate practitioners exhibit superior balance compared to untrained individuals or athletes from non-combat sports [16]. These studies emphasize that elite karatekas develop highly efficient Center of Pressure (CoP) displacement strategies, relying heavily on refined proprioceptive feedback and visual integration to maintain stability during high-speed technical executions [17]. Building upon this multiparametric framework, the authors of the present study previously contributed to the functional profiling of elite karatekas by investigating the discipline-specific functional adaptations between kumite and kata practitioners [18]. Utilizing a combination of wearable inertial sensors and optical systems, that study demonstrated that elite karatekas possess significantly superior joint mobility, postural stability, and jumping capabilities compared to non-athlete controls. Furthermore, it highlighted how the distinct technical demands of kumite and kata induce divergent neuromuscular and kinematic profiles, reinforcing the absolute necessity for sport-specific assessment protocols.

Despite the rigorous quantification of these physiological and biomechanical traits, the integration of sex and sex-specific variables into these functional profiles is surprisingly sparse. Recent literature, such as scoping reviews exploring the influence of sex dynamics on women’s experiences in martial arts [19], clearly highlights that female participation and athletic development are shaped by distinct socio-cultural, psychological, and physiological factors. However, while the sociological and psychological dimensions of sex in martial arts are beginning to be addressed, a significant gap remains in the literature investigating purely biomechanical and functional aspects. Historically, the majority of posturographic and neuromuscular studies in combat sports have either focused exclusively on male cohorts or aggregated male and female data, thereby masking potential sex-related functional dimorphisms [16,18,20,21,22].

However, accurately delineating these sex-specific kinematic and physiological profiles is of paramount importance for the optimization of training methodologies and the development of targeted injury prevention strategies. As early as 2012, Chaabène et al. [4] emphasized the critical need to establish specific normative data for both male and female elite karatekas to properly tailor physical conditioning. This necessity has been strongly reiterated in recent literature; a systematic review by Gaweł et al. [12] underscores that understanding distinct physical and physiological profiles, strictly differentiated by sex, is essential for establishing definitive performance benchmarks in elite WKF athletes. Specifically, defining sex-specific normative data for key parameters such as hamstring flexibility, speed development capacity, and aerobic endurance is deemed crucial not only for optimizing technical and tactical performance, but also for designing and implementing targeted injury prevention protocols. Furthermore, the relevance of exploring sex-specific biomechanical adaptations is corroborated by recent evidence in other martial arts. For instance, a recent study by Góra et al. [23] investigating Taekwondo athletes demonstrated significant sex-related differences in the biomechanics and impact strength of turning and side kicks. The same authors in another similar study [24] highlighted significant gender differences not only in absolute lower limb strength, but also in the underlying force generation strategies, such as the optimal use of effective mass during high-impact kicking techniques. Such findings reinforce the notion that generalized, mixed-sex functional models are inadequate for elite combat sports, where optimal power generation, postural stability, and technical execution are intricately linked to sex-specific physiological and kinematic characteristics.

Crucially, what is currently lacking in the existing literature is a simultaneous, high-resolution comparison of joint-specific kinematics at maximum velocities, phase-specific temporal metrics during jumping such as eccentric vs. concentric phase durations, and visual dependency in single-leg stance between elite male and female karatekas. The present study directly addresses this gap. By employing a comprehensive, multiparametric approach, this research advances the field beyond basic performance outcomes. It aims to objectively quantify these previously overlooked functional dimorphisms, shedding light on the distinct, sex-specific neuromotor and postural strategies utilized by elite athletes.

Therefore, the primary aim of this study was to conduct a comprehensive, multiparametric assessment to identify sex-specific biomechanical and functional adaptations in elite karate athletes. Specifically, we sought to compare lower-limb explosive power, specific joint kinematics, and visual-dependent postural control between male and female international-level karatekas.

Based on the existing literature and the physiological dimorphisms between sexes, we formulated three main hypotheses. First, we hypothesized that male athletes would exhibit superior neuromuscular explosive power, resulting in greater flight times during vertical jump performances. Second, we hypothesized that female athletes would demonstrate greater lower-limb joint mobility. Finally, regarding postural control, we hypothesized that while baseline stabilometric performance under normal visual conditions might present sex-related differences, the reliance on visual feedback for maintaining balance would be uniform across both sexes, reflecting a shared, sport-specific postural strategy developed at the elite level.

## 2. Materials and Methods

### 2.1. Participants

The study included 28 elite, international-level karatekas from the Italian national team, all possessing a minimum of 15 years of training experience. The male group consisted of 14 karatekas (MK) (age = 24 ± 6 years, height = 173.4 ± 7.6 cm, body mass = 74.8 ± 9.4 kg) and the female group consisted of another 14 karatekas (FK) (age = 25 ± 5 years, height = 165.4 ± 5.6 cm, body mass = 60.8 ± 4.4 kg). Importantly, the distribution of competitive specializations was perfectly identical between the two groups. Both the MK and FK groups included 4 kata athletes and 10 kumite athletes. This specific proportion exactly mirrors the composition of the national team rosters: the kata squad comprises 3 starters and 1 reserve per sex (n = 4), whereas the kumite squad features 5 weight categories with 2 athletes summoned per category (n = 10). A balanced design is generally recommended in experimental research to ensure that potential confounding factors are equally distributed across groups, thereby reducing their influence on the primary comparison [25]. The sample featured highly decorated athletes across various weight and skill categories, including Italian, European, and World champions, as well as Olympic medalists. Their training regimen comprised two daily sessions, six days a week. None of the participants had sustained any time-loss musculoskeletal injuries within the previous two years, defined as injuries that prevented regular training or competition. After being fully informed about the study’s purpose, all subjects provided written informed consent in compliance with the ethical principles of the 1964 Declaration of Helsinki. The research protocol was approved by the Ethical Committee of the University of Tuscia on 22 December 2024 (approval no. 22122024).

### 2.2. Experimental Setup and Protocol

The experimental protocol utilized a combination of two sensor systems, which are OptoGait and GyKo (Microgate S.r.l., Bolzano, Italy), alongside a dual-camera video recording setup.

The OptoGait system allowed for the acquisition of spatiotemporal parameters. This device comprises 1 m transmitting and receiving bars placed at ground level, each equipped with 96 LED diodes. Operating at a sampling frequency of 1 kHz, the system detects foot flight and contact times with a high precision of 1 ms. Data were recorded via OptoGait software (Version 1.15.2.0). This system is widely validated and commonly employed for human movement assessment, particularly for gait and jump analyses [26,27,28].

The GyKo inertial sensor allowed for the acquisition of kinematic data. The Gyko is a single wearable Inertial Measurement Unit (IMU; dimensions: 50 × 70 × 20 mm, mass: 35 g). The IMU integrates a triaxial accelerometer (full-scale range: ±2 g to ±16 g), a triaxial gyroscope (±2000 °/s), and a triaxial magnetometer (±4800 µT). To prevent motion artifacts while ensuring participant comfort, the sensor was firmly attached to the relevant body segment using a customized semi-elastic belt with a magnetic mount. Data were transmitted via Bluetooth to a dedicated PC and processed at a sampling rate of 500 Hz using GyKoRepower software (v. 1.2.4.0). The GyKo IMU is a well-established tool for evaluating motor tasks such as jumps [29], muscle strength [30], and stability [31,32], demonstrating high reliability for both angular kinematics [33] and postural control metrics [34].

Finally, to monitor protocol compliance, two Nexigo N660P (Nexight INC, Beaverton, OR, USA) webcams were positioned 3 m away from the participant, capturing the frontal and sagittal (lateral) planes. Video recordings were temporally synchronized with the sensor data acquisition. All trials were subsequently reviewed, and any incorrectly executed tasks were excluded from the final data analysis.

The experimental design comprised three specific tasks aimed at assessing whole-body joint mobility, single-leg (monopodalic) stability, and jumping performance. The selected exercises were developed in close collaboration with the technical staff of the Italian national karate team to ensure alignment with sport-specific requirements. To closely replicate standard training conditions, all tests were performed barefoot directly on the tatami (karate mat).

The entire protocol, with the execution order of the distinct tests randomized for each participant, required approximately 45 min, and all subjects successfully completed all tests.

#### 2.2.1. Joint Mobility Assessment

For the joint mobility tasks, participants were instructed to perform specific movements of the shoulder, hip, and ankle joints. Each movement aimed to achieve the maximum RoM at the subject’s preferred velocity, while strictly minimizing compensatory motions from other body segments. Additionally, shoulder movements and hip flexion were also executed at maximum velocity (VMAX). Each specific movement was performed three times.

Kinematic data were acquired using the GyKo, which was securely attached to the moving body segment corresponding to the evaluated joint to record linear acceleration and angular velocity. For these tasks, the accelerometer’s full-scale range was adjusted to ±2 g, and the OptoGait system was not utilized.

**Figure 1:**
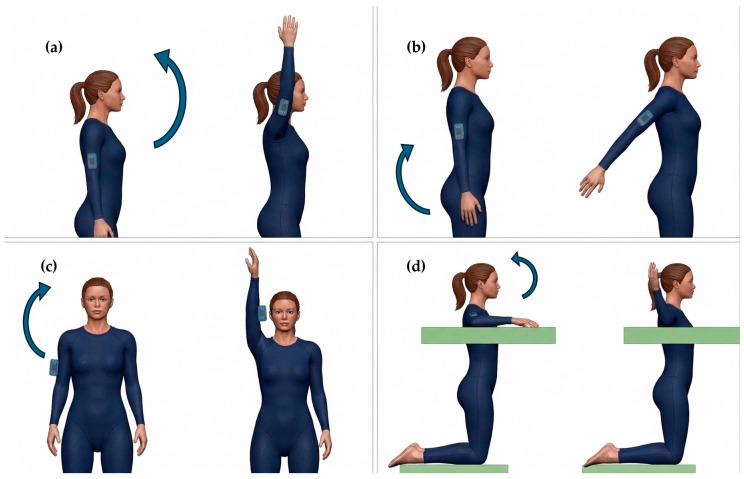
Mobility of shoulder: sensor positioning and movement direction for the flexion (**a**), extension (**b**), abduction (**c**) and external rotation (**d**) task.

The shoulder mobility assessment comprised four distinct movements: flexion, extension, abduction, and external rotation, performed bilaterally (both sides) under both preferred and maximum velocity conditions:

- Flexion, extension, and abduction: Participants stood barefoot on the tatami, initiating the movements from a neutral standing position with arms resting alongside the torso (Figure 1a–c). During flexion and extension, internal and external rotations were strictly restricted; conversely, natural physiological coupled motions were permitted during abduction. For these three movements, the IMU was positioned on the arm, 15 cm distal to the shoulder’s center of rotation.
- External rotation: participants began with the shoulder abducted at 90°, resting their forearm and hand on a flat support surface (Figure 1d). For this specific task, the sensor was attached to the forearm, 15 cm distal to the elbow joint.

Hip joint mobility was evaluated through three specific functional tasks.

**Figure 2:**
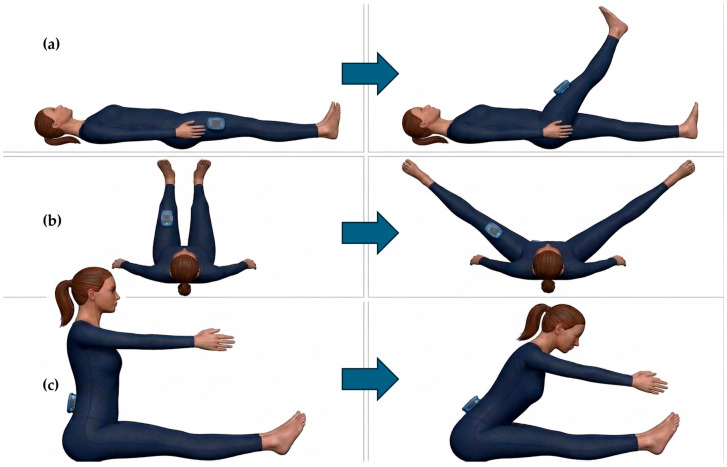
Mobility of hip: positioning of the sensor and movement direction for flexion (**a**), split (**b**), and sit and reach (**c**) task.

- Supine Hip Flexion: Participants started in a supine position with both legs fully extended on the ground. They were instructed to perform a unilateral hip flexion while maintaining full knee extension (Figure 2a). This task was performed bilaterally (both sides) and was the only hip movement executed at both preferred and maximum velocities.
- Wall Split (Bilateral Abduction): Participants lay supine with their trunk flat on the floor and legs resting vertically against a wall, maintaining a 90° hip flexion. From this starting position, they performed a simultaneous bilateral hip abduction (Figure 2b). Because a single IMU was utilized, this protocol was repeated twice to allow separate data acquisition for each leg.
- Sit-and-Reach: This task assessed forward trunk mobility and further hip flexion. Participants started in a long-sitting position with legs fully extended on the floor and performed a forward reaching motion with their trunk and arms (Figure 2c).

For the supine hip flexion and wall split movements, GyKo was positioned on the anterior thigh, 15 cm proximal to the knee’s center of rotation (Figure 2a,b). Conversely, during the sit-and-reach test, the sensor was securely attached to the participant’s lower back at the level of the L4 vertebra (Figure 2c).

Ankle joint mobility was assessed through two distinct functional movements, both executed exclusively at the participant’s preferred, self-selected velocity.

**Figure 3:**
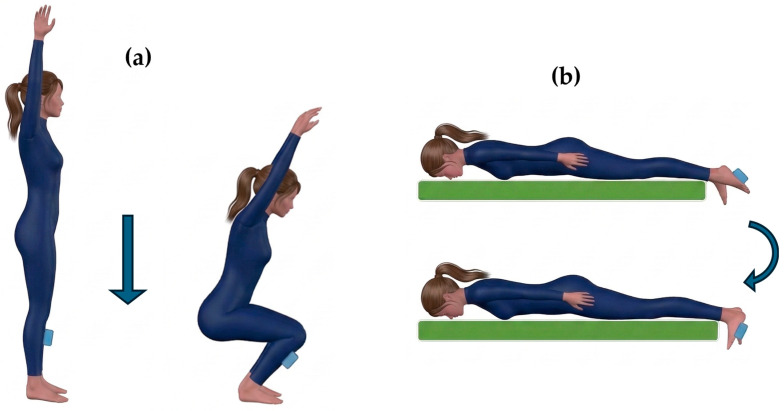
Mobility of ankle: positioning of the sensor and movement direction for squat (**a**) and dorsiflexion (**b**) task.

- Overhead Squat (Closed-Kinetic-Chain Dorsiflexion): The first task evaluated ankle flexion driven by tibial advancement (leg rotation over the foot) during an overhead squat (Figure 3a). Since a single IMU was utilized, the squat movement was performed twice to allow independent data acquisition for each leg.
- Prone Ankle Flexion: The second task consisted of an active ankle flexion (dorsiflexion) isolated to foot rotation. Participants lay prone on an examination couch with their feet extending beyond the edge, starting from a fully extended (plantarflexed) resting position (Figure 3b). This movement was performed bilaterally.

Sensor positioning was adapted according to the specific mechanics of each task. During the prone flexion movement, GyKo sensor was secured to the plantar fascia of the evaluated foot (Figure 3b). Conversely, for the overhead squat task, the IMU was placed on the anterior shank (tibia) at a distance of 10 cm distal to the knee joint line (Figure 3a). Prior to the execution of each dynamic task, a static baseline acquisition was recorded while the sensorized body segment was held in a resting state. This resting posture corresponded to the specific starting position of each individual test.

#### 2.2.2. Postural Stability Assessment

To evaluate postural stability, a single-leg stance test was implemented. This protocol was adapted from the traditional One-Leg Standing Balance test [35], with specific modifications to the arm and raised-leg positions dictated by the national karate coaching staff to better reflect sport-specific biomechanical demands.

Participants were instructed to stand barefoot on their dominant leg for 15 s. The contralateral limb was positioned with the hip, knee, and ankle all flexed at 90°. Concurrently, participants were required to keep their hands crossed behind their head. The test was conducted under two visual conditions: Eyes Open (EO) and Eyes Closed (EC). During the EO trials, subjects were asked to fixate on a visual target positioned on a wall 5 m away. A trial was deemed successfully completed if the participant maintained the posture for the full 15 s without the raised foot touching the ground and, during the EC condition, without opening their eyes. Each condition was recorded once, following an initial familiarization period.

**Figure 4:**
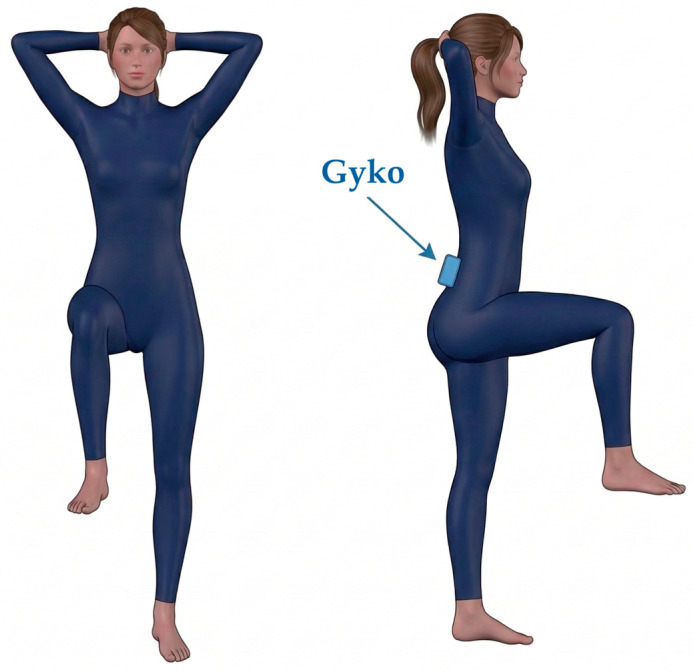
Postural Stability Assessment: positioning of the subject and sensor.

Postural stability was quantified using GyKo, securely attached to the participant’s lower back at the L4 vertebral level to measure the linear acceleration and angular velocity of the pelvis (Figure 4). For each subject, the vertical distance from the center of the sensor to the ground was recorded prior to the test. Prior to the execution of each dynamic task, a static baseline acquisition was recorded while the sensorized body segment was held in a resting state. This resting posture corresponded to the specific starting position. Consistent with the mobility tasks, the IMU accelerometer’s full-scale range was set to ±2 g, and the OptoGait system was not active during this balance assessment.

#### 2.2.3. Jumping Performance Assessment

The evaluation of explosive power and jumping ability comprised three distinct tests: the Squat Jump, the Countermovement Jump, and a 15 s Repeated Countermovement Jump [18,28]. All trials were executed barefoot directly on the tatami.

For all jumping tasks, participants started from an upright standing position with their feet shoulder-width apart. To isolate lower-limb power and eliminate arm-swing assistance, subjects were instructed to keep their hands firmly placed on their iliac crests throughout the entire duration of the jumps. Participants were encouraged to jump as high as possible, with no imposed restrictions on the knee flexion angle during the preparatory phase.

- SJ: Participants descended into a semi-squat position, maintained this isometric stance for two seconds to dissipate elastic energy, and then executed an explosive upward concentric phase.
- CMJ and RCMJ: Participants performed a rapid downward (eccentric) countermovement immediately followed by the upward (concentric) propulsive phase, without any pause at the lowest point [29].

Both the SJ and single CMJ were performed three times independently, whereas the 15 s RCMJ was executed only once. To mitigate the effects of neuromuscular fatigue, a strict 2 min recovery period was enforced between all jump trials [18,28].

**Figure 5:**
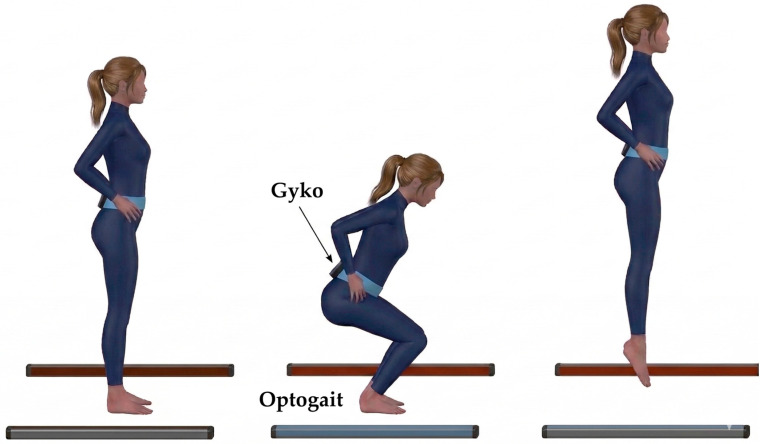
Jumping Performance Assessment: subject and sensor position and sequence of movements.

Kinematic data were gathered using GyKo, securely attached to the participant’s lower back at the L4 vertebral level to capture the linear acceleration and angular velocity of the pelvis. Prior to the execution of each dynamic task, a static baseline acquisition was recorded while the sensorized body segment was held in a resting state. This resting posture corresponded to the specific starting position. Given the high-impact nature of these dynamic tasks, the IMU accelerometer’s full-scale range was increased to ±16 g. Concurrently, spatiotemporal parameters were recorded using the OptoGait system. The OptoGait transmitting and receiving bars were positioned directly on the tatami, set at a distance of 2.5 m apart (Figure 5).

### 2.3. Data Analysis

Data acquired during the initial static trial were utilized to align the sensor axes with the absolute global reference frame. Subsequently, for all executed tests, the raw linear accelerations and angular velocities recorded by GyKo were processed using a sensor fusion algorithm incorporating a Mahony complementary filter [36]. For reproducibility, the filter parameters were set to standard default values suitable for short-duration biomechanical tasks: a proportional gain (K_p_) of 1.0 was used to correct orientation errors via accelerometer updates, while the integral gain (K_i_) was set to 0.0, as significant gyroscope bias drift was not anticipated during the brief recording windows of the selected movements.

Regarding the mobility tasks performed at the subject’s preferred, self-selected velocity, the maximum angular displacement along the examined axis was denoted as α. This kinematic variable was calculated as the peak difference between the angle measured during the movement execution and the baseline angle recorded at the starting rest position. Additionally, the average angular velocity of the performed movement was defined as $\dot{\alpha }$. For the subset of tasks specifically executed at maximum velocity (i.e., all shoulder movements and supine hip flexion), these identical kinematic metrics were computed and respectively denoted as α_VMAX_ and $\dot{\alpha }$_VMAX_. Since each single measurement was repeated three times, the average value of the three tests was used for the subsequent statistical analysis.

**Figure 6:**
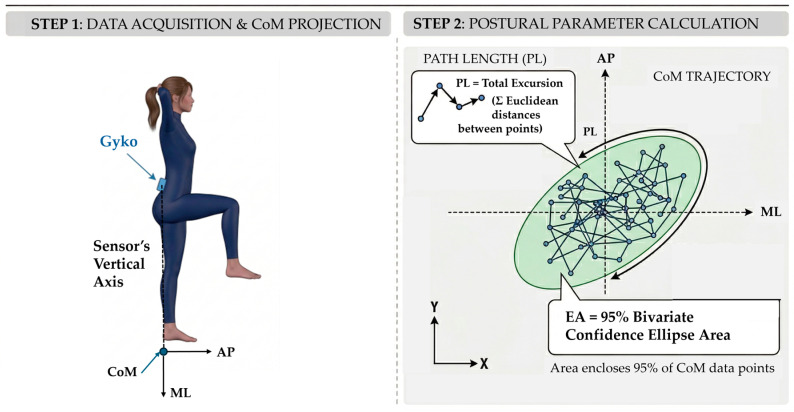
Schematic representation of the methodological protocol for CoM quantification. Step 1 illustrates sensor data acquisition and the geometric projection for ground CoM estimation. Step 2 describes the calculation of posturographic parameters on the Antero-Posterior (AP) and Medio-Lateral (ML) axes: Path Length (PL), i.e., the total trajectory excursion, and the 95% Confidence Ellipse Area (EA).

Postural stability was quantified by projecting the sensor’s vertical axis onto the horizontal ground plane. This projection was geometrically derived using the initial sensor-to-floor height, measured with a metric tape prior to the test. The resulting two-dimensional coordinate was adopted as a sensor-based proxy of CoM displacement, allowing for the extraction of its anteroposterior (AP) and mediolateral (ML) sway trajectories. To evaluate postural control, standard posturographic indices were calculated [37,38]. Specifically, the Path Length (PL) and the 95% Confidence Ellipse Area (EA) were computed based on the mathematical formulations detailed in [38]. PL represents the total excursion of the CoM, calculated as the sum of the Euclidean distances between consecutive CoM data points. EA was defined as the area of the 95% bivariate confidence ellipse, which statistically encloses approximately 95% of the recorded CoM trajectory data points. A representative scheme is shown in Figure 6.

Furthermore, the Romberg Index (RI) was calculated for both PL and EA to assess the impact of visual deprivation [37]. The RI was computed as the ratio of the stability parameter measured under Eyes Closed (EC) to that under Eyes Open (EO) conditions. An RI value approaching 1.0 denotes excellent postural adaptation and a lower reliance on visual feedback to maintain balance [38].

Regarding the jump tests, take-off and touchdown instants were detected using the OptoGait photoelectric system. Simultaneously, the vertical linear acceleration acquired from the GyKo IMU was numerically integrated to derive the vertical linear velocity, enabling the precise identification of the jumps’ eccentric and concentric phases.

Phase Definitions:

- The eccentric phase was defined as the time interval from the initiation of the downward movement to the instant of zero velocity (i.e., the transition point where the subject reverses direction to push upwards).
- The concentric phase was defined as the time interval from the end of the eccentric phase (zero velocity) to the instant of take-off.

Extracted Variables:

- For the SJ, Flight Time (FT) and Concentric Phase Time (CPT) were calculated.
- For the CMJ, the Eccentric Phase Time (EPT) was additionally computed. For both single SJ and CMJ, these metrics were averaged across the three recorded repetitions for each participant, as no trials were excluded following the video compliance review.
- For the RCMJ, all the aforementioned parameters were calculated, alongside the Contact Time (CT). Specifically, for FT and CT, both the maximum (FT_MAX_ and CT_MAX_) and mean (FT_MEAN_ and CT_MEAN_) values over the 15 s period were extracted; whereas for EPT and CPT, only the mean (EPT_MEAN_ and CPT_MEAN_) values over the 15 s were considered.

Similarly to the joint mobility tests, also for SJ and CMJ, since each single measurement was repeated three times, the average values of the three tests was therefore used for the subsequent statistical analysis.

### 2.4. Statistical Analysis

Statistical analysis was performed using SPSS version 28.0 (IBM Corp., Armonk, NY, USA). Descriptive statistics were calculated for all evaluated parameters. To ensure appropriate data representation, variables are reported as Mean ± Standard Deviation (SD) when normally distributed, and as Median and Interquartile Range (IQR: 25th–75th percentiles) when the normality assumption was not met.

The assumption of normality was assessed for each variable and within each group (males and females) using the Shapiro–Wilk test. Homogeneity of variances was evaluated via Levene’s test. For variables demonstrating a normal distribution, an independent samples *t*-test was utilized to assess sex differences. In cases where Levene’s test indicated unequal variances, Welch’s *t*-test was applied.

Conversely, for variables that significantly deviated from a normal distribution, the non-parametric Mann–Whitney *U* test was employed to compare the male and female groups. The level of statistical significance for all tests was set a priori at *p* < 0.05.

## 3. Results

A preliminary within-group analysis was performed to evaluate potential bilateral differences between left and right limbs. Paired samples *t*-tests were used for normally distributed variables, while Wilcoxon signed-rank tests were applied for non-normally distributed data. No statistically significant differences were observed across all evaluated parameters (all *p* > 0.05), with small effect sizes (Cohen’s *d* < 0.30), supporting the decision to pool data from both sides for subsequent analyses.

### 3.1. Joint Mobility Assessment

Table 1 presents the mean angular displacements and standard deviations (SD) computed during the joint mobility tasks executed at the self-selected preferred velocity, alongside the corresponding statistical outcomes. Data from the right and left sides were pooled for the main analysis, as a preliminary within-group evaluation revealed no significant bilateral differences.

Regarding the maximum angular displacement α, significant differences between sexes were observed in only two specific movements, both supported by large effect sizes. In particular, MK during shoulder extension demonstrated a significantly greater range of motion compared to females (84.2 ± 6.5° vs. 74.9 ± 13.1°, respectively; *p* = 0.039, *d* = 0.90); conversely, during Wall Split FK exhibited a significantly wider bilateral abduction angle (81.6 ± 6.7° vs. 72.1 ± 6.6° for males; *p* = 0.002, *d* = 1.43). No further statistically significant differences in joint excursion were detected between MK and FK for the remaining shoulder, hip, and ankle mobility tasks. When analyzing the kinematic execution speed, the mean angular velocity $\dot{\alpha }$ revealed no significant differences between MK and FK across any of the assessed joints and movements. This indicates a highly similar execution speed profile between the two groups during self-paced tasks.

Table 2 presents the mean angular displacements and standard deviations (SD) recorded during the joint mobility tasks executed at maximum velocity, along with the corresponding statistical outcomes. As a preliminary within-group analysis revealed no significant bilateral differences, data from the right and left sides were pooled for the primary evaluation.

In contrast to the results obtained at the preferred velocity, the analysis of the maximum angular displacement revealed no statistically significant differences between MK and FK across any of the evaluated shoulder and hip movements. However, significant between-group differences emerged when analyzing the execution speed. Specifically, MK demonstrated a significantly higher maximum angular velocity during sagittal plane shoulder movements compared to their female counterparts. This was evident during both shoulder flexion (582.7 ± 89.4 °/s for MK vs. 472.0 ± 92.4 °/s for FK; *p* = 0.007, *d* = 1.22) and shoulder extension (399.5 ± 56.4 °/s for MK vs. 341.6 ± 73.7 °/s for FK; *p* = 0.041, *d* = 0.88) with both movements exhibiting a large effect size. No significant sex differences in maximum angular velocity were detected for shoulder abduction, shoulder external rotation, or hip flexion.

### 3.2. Postural Stability Assessment

**Figure 7:**
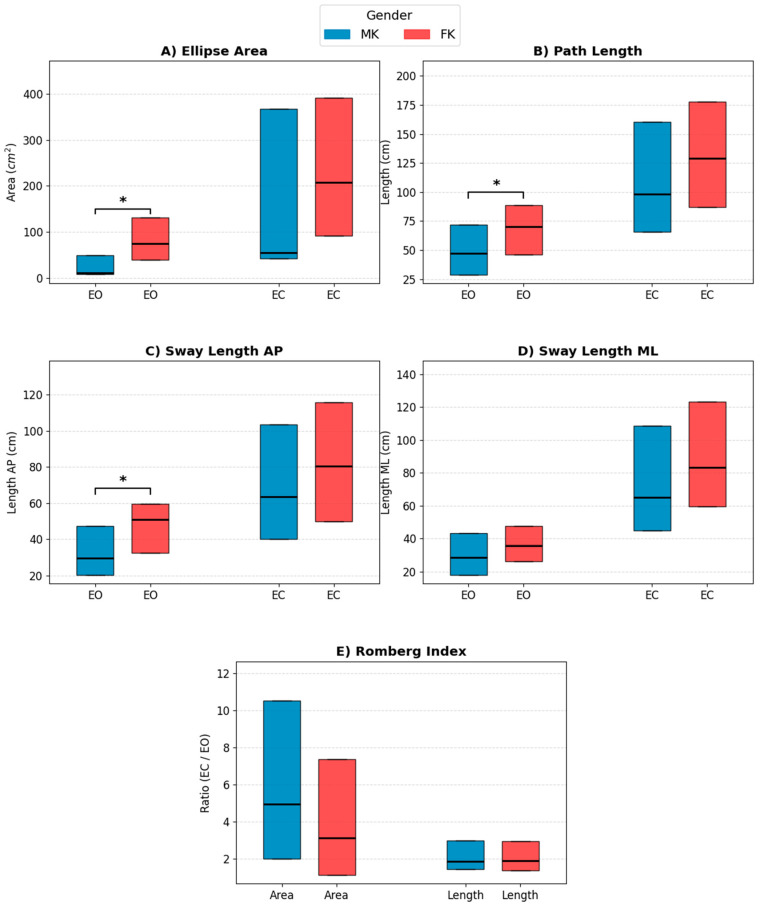
Comparison of postural stability parameters between MK (blue) and FK (red) karatekas under eyes open (EO) and eyes closed (EC) conditions. Boxplots represent the median and interquartile range (IQR) for: (**A**) Ellipse Area; (**B**) Path Length; (**C**) Antero-Posterior Sway Length (AP); (**D**) Medio-Lateral Sway Length (ML); and (**E**) Romberg Index (calculated for both Area and Length). The asterisk (*) denotes a statistically significant difference between sexes (*p* < 0.05).

Due to the non-normal distribution of the data, as confirmed by the Shapiro–Wilk test, sex differences in postural stability were analyzed using the non-parametric Mann–Whitney *U* test. Consequently, the relevant postural stability parameters are reported as Median and Interquartile Range (IQR: 25th–75th percentiles), as presented in Figure 7 and Table 3.

Under the EO condition, significant sex-specific differences emerged in the spatial dimensions of balance control, supported by moderate-to-large effect sizes. FK exhibited significantly greater postural oscillations compared to males, as evidenced by a larger Ellipse Area (*p* = 0.006, *r* = 0.52) and a greater total Path Length (*p* = 0.033, *r* = 0.40).

Strikingly, under the EC condition, visual deprivation led to a substantial increase in overall postural instability for both groups, causing the previously observed sex-related differences to completely disappear. Without visual feedback, males and females demonstrated statistically comparable stabilometric performances for both the Ellipse Area (*p* = 0.102, *r* = 0.31) and the Path Length (*p* = 0.185, *r* = 0.25).

This uniform reliance on visual input was further confirmed by the Romberg Index. The analysis revealed no significant differences between males and females in the Romberg Index for either the Ellipse Area (*p* = 0.355, *r* = 0.17) or the Path Length (*p* = 0.962, *r* = 0.01).

A further aspect that emerged from the analysis is the greater inter-subject variability observed within the female group (FK), particularly with regard to joint movement speeds and stabilometric parameters, as highlighted by the width of the interquartile ranges (IQR) and the dispersion of the data in the boxplots.

### 3.3. Jumping Performance Assessment

**Figure 8:**
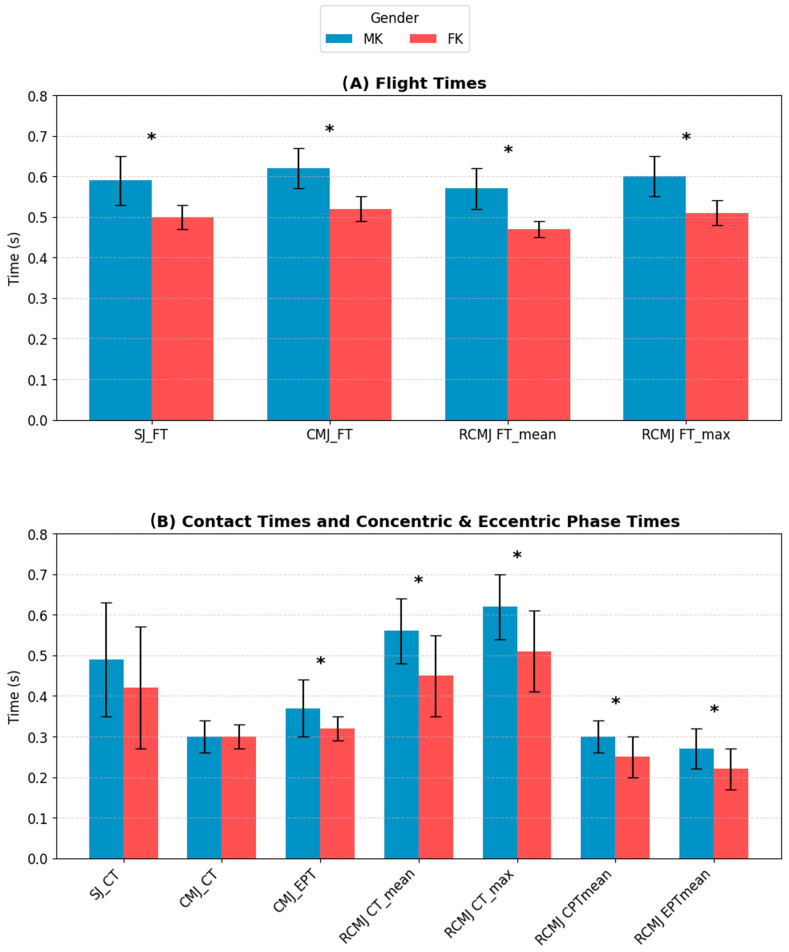
Comparison of jump performance between MK (blue) and FK (red) karatekas. Bar charts represent the Mean ± Standard Deviation (SD) for: (**A**) Flight Times (FT) during Squat Jump (SJ), Countermovement Jump (CMJ), and 15 s Repeated CMJ (RCMJ); (**B**) Contact Times (CT), Concentric Phase Times (CPT), and Eccentric Phase Times (EPT) across the different jump protocols. Asterisks (*) indicate a statistically significant difference between sexes (*p* < 0.05).

The descriptive statistics (Mean ± SD) and the independent samples *t*-test results for jump performance are summarized in Figure 8 and Table 4.

Analysis of the jump tests revealed a clear sex-specific dimorphism, supported by very large effect sizes. MK demonstrated significantly higher FT compared to females in both the SJ (0.59 ± 0.06 s vs. 0.50 ± 0.03 s; *p* < 0.001, *d* = 1.90) and the CMJ (0.62 ± 0.05 s vs. 0.52 ± 0.03 s; *p* < 0.001, *d* = 2.43). Conversely, no significant sex differences were observed in the CPT for either the single SJ (*p* = 0.228, *d* = 0.48) or the single CMJ (*p* = 0.774, *d* = 0.00), although MK exhibited a significantly longer EPT in the CMJ (0.37 ± 0.07 s vs. 0.32 ± 0.03 s; *p* = 0.043, *d* = 0.93). The significant difference in FT output was consistent throughout the 15 s RCM test, where MK exhibited significantly greater FT_MEAN_ and FT_MAX_ (*p* < 0.001 for both parameters, with *d* = 2.63 and *d* = 2.18 respectively). Furthermore, the temporal phase analysis indicated that MK had significantly longer ground CT during the RCMJ (CT_MEAN_ 0.56 ± 0.08 s vs. 0.45 ± 0.10 s; *p* = 0.006, *d* = 1.21; CT_MAX_ 0.62 ± 0.08 s vs. 0.51 ± 0.10 s; *p* = 0.005, *d* = 1.21), as well as significantly extended durations in both the CPT_MEAN_ (*p* = 0.010, *d* = 1.10) and EPT_MEAN_ (*p* = 0.016, *d* = 1.00) of the repeated jumps compared to their female counterparts, with all temporal metrics exhibiting large effect sizes.

## 4. Discussion

The primary aim of this study was to conduct a multiparametric assessment to identify sex-specific biomechanical and functional adaptations in elite karate athletes. The results obtained largely confirm our initial hypotheses.

Kinematic analysis revealed two distinct and complementary functional profiles between the sexes, highlighting specific adaptation strategies to the technical gesture. On the one hand, the FK demonstrated significantly greater flexibility at the coxofemoral level, as evidenced by the greater bilateral hip abduction angle. This greater hip flexibility observed in female athletes is in line with what was highlighted by Chaabène et al. [4] and Hariri [6], who identified the wide RoM of the hip as an essential biomechanical prerequisite for the effectiveness of high roundhouse kicks (e.g., Mawashi-Geri). Furthermore, in accordance with recent evidence in combat sports [23], such hypermobility could represent a specific female compensatory strategy to maximize distal kinetic energy by reducing antagonistic resistances. On the other hand, MK demonstrated a clear advantage in the explosiveness of the upper kinematic chain. Although shoulder motion amplitudes at self-selected speed did not differ significantly between groups, execution at maximum speed revealed a different picture: males generated significantly higher maximum angular velocities, particularly in shoulder flexion and extension movements. The higher peak angular velocity of the shoulder recorded in the male group supports and contextualizes the recent findings of Goethel et al. [8], who identified upper limb acceleration as one of the main indicators of neuromuscular control for the execution of Gyaku-Tsuki. This finding aligns with the work of Loturco et al. [39], who demonstrated that punch acceleration represents the most discriminating biomechanical variable for competitive success in elite karate. Consequently, the male ability to generate significantly higher peak angular velocity likely translates into a potential kinematic advantage in the impact phase of linear punch techniques.

Analysis of jumping performance revealed marked sex-related differences, highlighting how male athletes generate significantly superior neuromuscular power. Specifically, the male group achieved significantly longer FT than the female group in all protocols examined, including the SJ, CMJ, and RCMJ. This finding reflects the known physiological superiority of males in the expression of explosive strength and in the greater efficiency of the stretch-shortening cycle. As highlighted in the literature by McMahon et al. [40], male athletes are able to generate significantly greater net impulse and peak force during the propulsive phase of the jump, factors directly related to the increase in flight time. A particularly relevant biomechanical aspect emerges from the analysis of time phases during the RCMJ. Although males maintained significantly higher FT for the entire 15 s test, they simultaneously recorded significantly longer mean and maximum CT than female athletes. Specifically, this difference manifested itself in significantly extended durations in both the concentric and eccentric phases. This suggests the adoption of a specific and divergent neuromotor strategy between the two sexes. To express and maintain such high-power output over time, male athletes require a physiologically longer elastic loading (eccentric) and propulsive push-off (concentric) phase. One possible explanation is that female athletes might compensate for their lower absolute force output by optimizing joint stiffness and adopting a strategy characterized by greater ground reactivity and reduced contact times. This dynamic is consistent with sex differences in the use of the stretch-shortening cycle, where female athletes tend to favor a fast stretch-shortening cycle to maximize elastic efficiency at the expense of pure power [41]. In the specific context of kumite karate, where the speed of footwork and distance changes is fundamental, this dynamic takes on crucial applicative importance. As suggested by Roschel et al. [13], vertical jump performance is a strong predictor of combat effectiveness; therefore, female athletes’ enhanced ground reactivity could translate into a clear advantage in rapid evasive maneuvers or sudden distance closures, compensating for their lower absolute propulsive force in competitive situations.

The stabilometric analysis offered particularly innovative results regarding monopodalic balance control strategies, revealing unexpected dynamics. Under normal visual input conditions (EO), female athletes exhibited significantly greater postural sway than males, quantified by a higher Ellipse Area and Path Length. However, the most relevant methodological and functional aspect emerged with visual deprivation (EC). The removal of visual feedback caused a drastic and immediate decline in stability in both groups, bringing them to the same level of instability and completely eliminating the previously observed sex-specific differences (*p* > 0.05 for all spatial parameters). This phenomenon is supported by the analysis of the Romberg Index, which was statistically identical between males and females for both sway Area and Path Length. These results demonstrate that, despite starting from different baseline levels, upon reaching the international competitive elite, both men and women develop the same dependence on visual afferents to ensure correct postural control in single-leg stance. This evidence extends and contextualizes the findings of Gauchard et al. [16], who had already highlighted how high-level karate practitioners adopt highly specialized postural strategies, based on a strong visual anchoring to stabilize the sudden translations of the center of mass. This visual-dependent strategy represents a universal sport-specific adaptation in elite karate, which transcends biological sex differences. Furthermore, a further finding of considerable practical interest lies in the high inter-subject variability found within the female group, highlighted by the wide data dispersion (IQR) in the boxplots for stabilometric parameters. This marked heterogeneity suggests that, at the same competitive level, female athletes do not rely on a single “standard postural model” but draw on a much more diverse repertoire of compensatory neuromuscular strategies than males. This variability is largely justified by physiological dimorphisms: as demonstrated by Hu et al. [42], females intrinsically present greater levels of joint laxity and a different peripheral proprioceptive sensitivity. Consequently, to maintain optimal monopodalic balance, female athletes must rely more on continuous micro-adjustments and on different activation strategies of the stabilizing muscles of the core and ankle, thus explaining the increase in Ellipse Area and Path Length recorded in baseline conditions [43,44].

### Practical Implications and Limitations

Based on the differences identified, the results of this study suggest the need to move beyond standardized athletic training approaches in favor of sex-specific conditioning programs for elite karateka.

Specifically, for female athletes, training protocols might benefit from a greater focus on developing maximal and explosive lower limb strength to help mitigate the gap in absolute power. Furthermore, given the greater basic postural range and high intersubject variability observed in the female group, it could be highly beneficial to integrate advanced exercises for stability and deep proprioceptive control.

Conversely, for male athletes, programming may consider emphasizing joint mobility and flexibility routines, with particular attention to the coxofemoral region, to support the optimal range of motion required for high kick techniques. Likewise, to optimize reactivity in kumite footwork, males might benefit from reactivity-focused plyometric exercises, aimed at reducing the prolonged ground contact times seen during repeated jumps. Finally, for both sexes, the reliance on visual afferents suggests the potential usefulness of introducing visual perturbations and neurocognitive challenges during balance training to help stimulate somatosensory and vestibular compensatory strategies.

Despite the innovative nature of the multiparametric approach and the relevance of its practical implications, this study presents some methodological limitations that must be carefully considered. First, the sample size analyzed is numerically limited and no a priori power analysis was conducted. Future studies should aim to include larger sample sizes and adopt more advanced statistical approaches, such as multivariate models or ANCOVA, to account for potential covariates (e.g., body mass, training specialization, and hormonal factors) and further refine the understanding of sex-specific biomechanical adaptations in elite karate athletes. Given the multiparametric nature of the analysis and the number of comparisons performed, there is a potential increment of Type I error risk. Although effect sizes and confidence intervals were reported to support the interpretation of the results, findings with marginal statistical significance should be interpreted cautiously. However, it is essential to contextualize this finding: the participants represent a cohort of top-level athletes. Recruiting large cohorts from such small and inaccessible elite populations is inherently difficult; the sample size was therefore dictated by the availability of these particular subjects. While the ecological and qualitative value of the sample partially compensates for the quantitative limitation, we acknowledge that this restricts the statistical power of our findings and prevents us from drawing definitive conclusions. Second, for numerical reasons, the MK and FK included athletes specialized in both kata and kumite. As previously noted in the literature, these two disciplines induce divergent neuromuscular and postural adaptations. While the perfectly identical distribution of kata and kumite practitioners in both the male and female cohorts guarantees that discipline specialization does not act as a confounding variable for the main sex comparison, the aggregation of these athletes inevitably increased intra-group variability. This likely contributed to the high data dispersion observed, particularly in the stabilometric parameters. Unfortunately, due to the limited sub-sample sizes (n = 4 for kata), a statistically robust subgroup analysis comparing specific disciplines within each sex was not feasible in the present study. Finally, the menstrual cycle phase was not monitored in the female group at the time of testing. Since the literature widely recognizes that hormonal fluctuations (e.g., estrogen surges) can significantly alter ligament laxity and neuromuscular control, this uncontrolled variable may have influenced the hypermobility peaks and the greater stabilometric heterogeneity observed in female athletes. Future studies should specifically control for menstrual cycle phase to better isolate sex-related neuromuscular and postural adaptations.

## 5. Conclusions

This study provides a rigorously sex-specific biomechanical and functional profiling of elite karate athletes, going beyond the traditional methodological approach that tends to aggregate male and female data into generic models. The results suggest the existence of marked functional dimorphisms underlying high-level performance.

Male athletes excel in lower limb explosive power and maximum upper limb velocity, suggesting the adoption of propulsive neuromotor strategies based on longer contact times and elastic loading. Female athletes, on the other hand, compensate for their lower absolute strength by optimizing joint stiffness and leveraging significantly greater hip flexibility, essential for efficient kicking techniques. In the domain of postural control, the most significant finding concerns visual dependence: although women exhibit greater baseline heterogeneity and amplitude of oscillation, visual deprivation eliminates any stabilometric differences between the sexes. This suggests that elite karate athletes, regardless of sex, develop the same fundamental need for visual feedback to maintain monopodalic balance.

From an applied perspective, this scientific evidence indicates that the adoption of “unisex” training protocols may be inadequate for optimizing performance in contemporary karate. Physical trainers and medical staff are required to highly implement sex-specific conditioning programs. This methodological paradigm shift is essential not only to maximize technical efficiency and competitive success, but above all to provide objective tools, based on wearable sensors, aimed at targeted and sex-specific injury prevention in elite karate.

## Figures and Tables

**Table 1:** Mean values ± (standard deviations), *p*-values, Mean differences 95%CI and Effect Size d for α and $\dot{\alpha }$ obtained during the joint mobility test performed at the preferred velocity for the examined groups. MK and FK stand for male karatekas and female karatekas group, respectively. Significant differences are indicated in bold.

		α [°]					$\dot{\alpha }$ [°/s]				
		MK	FK	*p*-Value	Mean Diff [95%CI]	Cohen’s *d*	MK	FK	*p*-Value	Mean Diff [95%CI]	Cohen’s *d*
Shoulder	Flexion	179.3 ± 9.7	175.7 ± 10.8	0.403	[−4.37, 11.57]	0.35	270.6 ± 107.8	208.3 ± 143.4	0.238	[−36.26, 160.86]	0.49
	Extension	84.2 ± 6.5	74.9 ± 13.1	**0.039**	**[1.26, 17.34]**	**0.90**	174.5 ± 69.3	131.7 ± 124.3	0.302	[−35.39, 120.99]	0.43
	Abduction	171.1 ± 7.6	173.5 ± 8.6	0.476	[−8.71, 3.91]	0.30	286.4 ± 117.9	231.7 ± 174.9	0.373	[−61.19, 170.59]	0.37
	External-Rotation	106.8 ± 10.3	108.1 ± 9.3	0.749	[−8.93, 6.33]	0.13	209.8 ± 87.7	180.4 ± 141.9	0.543	[−62.29, 121.09]	0.25
Hip	Flexion	119.0 ± 13.0	119.4 ± 12.6	0.934	[−10.35, 9.55]	0.03	202.4 ± 59.0	165.7 ± 109.5	0.934	[−31.67, 105.07]	0.42
	Wall Split	72.1 ± 6.6	81.6 ± 6.7	**0.002**	**[−14.66, −4.34]**	**1.43**	122.8 ± 39.5	150.4 ± 58.8	0.185	[−66.51, 11.31]	0.55
	Sit and Reach	35.3 ± 7.9	38.7 ± 7.2	0.277	[−9.28, 2.48]	0.45	55.2 ± 24.4	54.9 ± 20.0	0.973	[−17.03, 17.63]	0.01
Ankle	Overhead squat	33.1 ± 7.3	31.3 ± 7.8	0.575	[−4.06, 7.66]	0.24	35.9 ± 15.0	30.5 ± 24.1	0.505	[−10.20, 21.00]	0.27
	Dorsiflexion	72.7 ± 6.1	75.7 ± 7.4	0.279	[−8.26, 2.26]	0.44	98.6 ± 46.7	82.8 ± 49.8	0.432	[−21.69, 53.29]	0.33

**Table 2:** Mean values ± (standard deviations), *p*-values, Mean differences 95%CI and Effect Size d for α_VMAX_ and $\dot{\alpha }$_VMAX_ obtained during the joint mobility test performed at the maximum velocity for the examined groups. MK and FK stand for male karatekas and female karatekas group, respectively. Significant differences are indicated in bold.

		α_VMAX_ [°]					$\dot{\alpha }$_VMAX_ [°/s]				
		MK	FK	*p*-Value	Mean Diff [95%CI]	Cohen’s *d*	MK	FK	*p*-Value	Mean Diff [95%CI]	Cohen’s *d*
Shoulder	Flexion	191.3 ± 9.2	191.4 ± 7.6	0.981	[−6.66, 6.46]	0.01	582.7 ± 89.4	472.0 ± 92.4	**0.007**	**[40.07, 181.33]**	**1.22**
	Extension	100.8 ± 6.2	97.4 ± 16.5	0.502	[−6.28, 13.08]	0.27	399.5 ± 56.4	341.6 ± 73.7	**0.041**	**[6.92, 108.88]**	**0.88**
	Abduction	177.4 ± 6.1	181.2 ± 7.6	0.186	[−9.15, 1.55]	0.55	531.5 ± 51.9	487.0 ± 97.0	0.169	[−15.94, 104.94]	0.57
	External-Rotation	116.9 ± 10.3	120.0 ± 7.5	0.411	[−10.10, 3.90]	0.34	527.6 ± 71.4	480.3 ± 56.4	0.091	[−2.69, 97.29]	0.74
Hip	Flexion	139.8 ± 14.3	139.4 ± 9.6	0.950	[−9.06, 9.86]	0.03	327.7 ± 51.8	305.6 ± 70.2	0.385	[−25.83, 70.03]	0.36

**Table 3:** Descriptive statistics and comparative analysis of stabilometric parameters between MK and FK groups under different visual conditions (EO, EC) and Romberg Index. Data are presented as Median (Interquartile Range) and Effect size *r*. Bold values indicate statistical significance.

	EO				EC				Romberg Index			
	MK	FK	*p*-Value	Effect Size *r*	MK	FK	*p*-Value	Effect Size *r*	MK	FK	*p*-Value	Effect Size *r*
Ellipse Area [cm^2^]	10.92 (7.34–48.61)	75.06 (39.12–130.88)	**0.006**	**0.52**	54.14 (42.31–367.59)	207.05 (91.91–391.09)	0.102	0.31	4.96 (1.99–10.52)	3.12 (1.13–7.35)	0.355	0.17
Path Length [cm]	47.01 (28.93–71.65)	70.35 (45.89–88.64)	**0.033**	**0.40**	98.10 (65.73–160.62)	128.80 (86.79–177.67)	0.185	0.25	1.86 (1.45–3.00)	1.91 (1.38–2.96)	0.962	0.01
Path Length AP [cm]	29.68 (20.33–47.11)	50.92 (32.43–59.67)	**0.018**	**0.45**	63.36 (40.01–103.32)	80.49 (49.97–115.76)	0.193	0.25				
Path Length ML [cm]	28.54 (18.01–43.13)	35.64 (26.20–47.79)	0.076	0.34	65.06 (44.90–108.59)	83.20 (59.40–123.38)	0.227	0.23				

**Table 4:** Mean values and standard deviations of the parameters related to the jumping tests. Parameters: Flight Time (FT), Concentric Phase Time (CPT), Eccentric Phase Time (EPT), and Contact Time (CT). MK and FK stand for male karatekas and female karatekas, respectively. Significant differences are indicated in bold.

		MK	FK	*p*-Value	Mean Diff [95%CI]	Cohen’s *d*
SJ	FT [s]	0.59 ± 0.06	0.50 ± 0.03	**0.000**	**[0.05, 0.13]**	**1.90**
	CPT [s]	0.49 ± 0.14	0.42 ± 0.15	0.228	[−0.04, 0.18]	0.48
CMJ	FT [s]	0.62 ± 0.05	0.52 ± 0.03	**0.000**	**[0.07, 0.13]**	**2.43**
	CPT [s]	0.30 ± 0.04	0.30 ± 0.03	0.774	[−0.03, 0.03]	0.00
	EPT [s]	0.37 ± 0.07	0.32 ± 0.03	**0.043**	**[0.01, 0.09]**	**0.93**
RCMJ	CT_MEAN_ [s]	0.56 ± 0.08	0.45 ± 0.10	**0.006**	**[0.04, 0.18]**	**1.21**
	CT_MAX_ [s]	0.62 ± 0.08	0.51 ± 0.10	**0.005**	**[0.04, 0.18]**	**1.21**
	FT_MEAN_ [s]	0.57 ± 0.05	0.47 ± 0.02	**0.000**	**[0.07, 0.13]**	**2.63**
	FT_MAX_ [s]	0.60 ± 0.05	0.51 ± 0.03	**0.000**	**[0.06, 0.12]**	**2.18**
	CPT_MEAN_ [s]	0.30 ± 0.04	0.25 ± 0.05	**0.010**	**[0.01, 0.09]**	**1.10**
	EPT_MEAN_ [s]	0.27 ± 0.05	0.22 ± 0.05	**0.016**	**[0.01, 0.09]**	**1.00**

## Data Availability

Data are available under the request to be referred to the corresponding authors.
